# Evaluating knowledge and skill in surgery clerkship during covid 19 pandemics: A single-center experience in Indonesia

**DOI:** 10.1016/j.amsu.2021.102685

**Published:** 2021-08-07

**Authors:** Eko Setiawan, Bambang Sugeng, Afridatul Luailiyah, Fadhli Rizal Makarim, Setyo Trisnadi

**Affiliations:** a Department of Surgery, Medical Faculty, Sultan Agung Islamic University, Semarang, 50164, Indonesia; b Department of Parasitology, Medical Faculty, Sultan Agung Islamic University, Semarang, 50164, Indonesia; c Department of Pathological Anatomy, Medical Faculty, Sultan Agung Islamic University, Semarang, 50164, Indonesia; d Department of Forensics and Medicolegal, Medical Faculty, Sultan Agung Islamic University, Semarang, 50164, Indonesia

**Keywords:** Surgery clerkship, Knowledge, OSCE, MCQs, Covid 19, OSCE, Objective Structured Clinical Examination, MCQs, Multiple-Choice Questions

## Abstract

**Backgrounds:**

Surgery clerkship for medical students has been changed in response to clinical exposure limitation due to this pandemic. This study aim to evaluate knowledge and skill of students in surgery clerkship in covid 19 pandemics.

**Methods:**

Cross-Sectional design comparing surgery clerkship before and during COVID-19.A total of 270 fourth and fifth-year medical students have enrolled in surgery clerkship from June 2019–October 2020 were selected for this study. Each student had completed education and training in the hospital for nine weeks in the rotation.

**Results:**

There is no significant difference in MCQs scores before and during the pandemic. However, a significant difference was found in OSCE scores.

**Conclusions:**

Combining virtual platforms and in-person clinical rotation is an effective surgery clerkship curriculum, particularly in pandemic covid 19. There are no different skill and knowledge results before and during the pandemic analyzed from MCQs and OSCE exam.

## Highlights

• An observational study compared knowledge and skills-based exam scores before and during the COVID-19 period in surgery clerkship.
• Knowledge score exam was found no difference before and during the COVID-19 period.
• The skills-based exam was found significantly better in students during the COVID-19 period.
• A combination of online and offline learning in surgery clerkship is an effective method in this pandemic era.

## 1 Introduction

The COVID-19 pandemic has a significant impact on the healthcare system and medical education, especially in clinical setting learning [1]. Surgery clerkship for medical students has been changed in response to clinical exposure limitation due to this pandemic to reach a level of competence in knowledge, hands-on, and clinical experience [2]. Whereas the student's clinical performance in surgery clerkship related to surgical knowledge, clinical competence, and reasoning [3]. Suspended rotation may implicate clinical competence, and a virtual platform may be an alternative to delivering surgery competence and experience [4]. Combining methods between virtual learning and surgical rotation with strict protocol may use as preferences. Therefore, workplace-based assessment of this combining surgery clerkship method is crucial, particularly in the skill and knowledge aspect.

## 2 Materials and methods

### 2.1 Study design

A cross-sectional study was conducted by comparing surgery clerkship before and during COVID-19 social distancing policies limiting medical student clinical participation on Sultan Agung Islamic University, Semarang, Indonesia. Before and during the COVID-19 period, knowledge and skills were measured by Objective Structured Clinical Examination (OSCE) and Multiple-Choice Questions (MCQs) scores. After that, profound data were taken by analyzing particular competencies used in OSCE and MCQs. A comparison of OSCE analysis such as suturing, fracture immobilization, and breast tumor examination was made in this study. Meanwhile, deep analysis for MCQs competence included a question in thyroid adenoma, urinary tract stones, colic, fracture, hernia, ileus, cholecystitis, burn injury, Buerger's disease, pneumothorax, and deep vein thrombosis.

### 2.2 Data sources

Institutional review board approval was obtained. A total of 270 fourth and fifth-year medical students have enrolled in surgery clerkship from June 2019–October 2020 were selected for this study. Each student had completed education and training in the hospital for nine weeks in the rotation. Students who joined clerkship on remedial status or did not complete the clerkship were excluded from this study. Original data were obtained from the Evaluation Committee of the Surgery Department.

### 2.3 Curriculum design

Before the COVID-19 period, the surgery rotation takes nine weeks and has several essential components. Each student has to complete a cycle in the emergency room, operating theatre, surgery ward, and outpatient clinic. Afterward, the students accomplish case-based discussion, journal reading, and textbook reading. Routine morning report discussions were held twice weekly, attended by the faculty member, supervisors, and students in the department hall. Every student has to take the night shift at least twice a week. After nine weeks, OSCE and MCQs were conducted to gain the final score of the clerkship. OSCE was done using an in-person exam method held in OSCE Center with five skill stations. Meanwhile, a hundred MCQs conducted using a computer-based test in a faculty designated location.

During COVID-19, the surgery rotation underwent alterations. The number of students was limited in each rotation. The clinical process in the emergency room was eliminated due to the high risk of infection. Night shifts were negated, and any offline meetings and activities were pushed down to fifty percent. Duty hours were being limited to 6 h per day. Case-based and routine morning discussion transitioned to an online platform using Zoom (Zoom Video Communications, Inc., San Jose, California) to discuss participants. OSCE was transformed into virtual using Zoom that connects students and examinators and used only three skill stations. However, MCQs still held a computer-based test in a specific location with at least 2 m of physical distance between computer workstations.

### 2.4 Statistical analysis

After examination scores were obtained, the averages and standard deviations were calculated. OSCE, MCQ, and in-depth analysis of competencies tested before and during the COVID-19 period were analyzed using Mann Whitney test.

## 3 Results

### 3.1 Participants

259 students were enrolled in this study, 115 students completed the rotation in COVID-19 social distancing settings from March 2020–October 2020. While 144 students completed the rotation in June 2019–February 2020, before the COVID-19 pandemic began.

### 3.2 OSCE and MCQ score

OSCE and MCQ scores were taken, mean and SD values were analyzed for each period. There is no significant difference in MCQ scores between the two groups. However, a significant difference was found in OSCE scores (Table 1).

**Table 1:** Comparison OSCE and MCQ scores before and during COVID-19 showed a significant difference in OSCE scores.

	Before COVID-19	During COVID-19	p value
	Mean ±SD	Mean ±SD	
OSCE Score	73.39±10.16	77.79±9.93	0.001
MCQ Score	66.69±14.23	65.36±12.67	0.605

In-depth Analysis of Selected Competencies in OSCE.

**Fig. 1:**
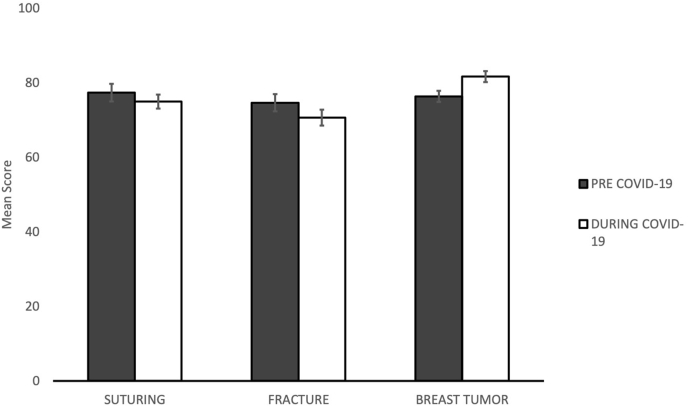
The mean score of each OSCE competency before and during covid 19 pandemics showed no significant difference except in breast tumors. In-depth Analysis of Selected Competencies in MCQ

The mean score of each competency tested was pooled and compared in the figure below (Fig. 1). Only breast tumor examination has a significant difference before and during the COVID-19 period.

**Fig. 2:**
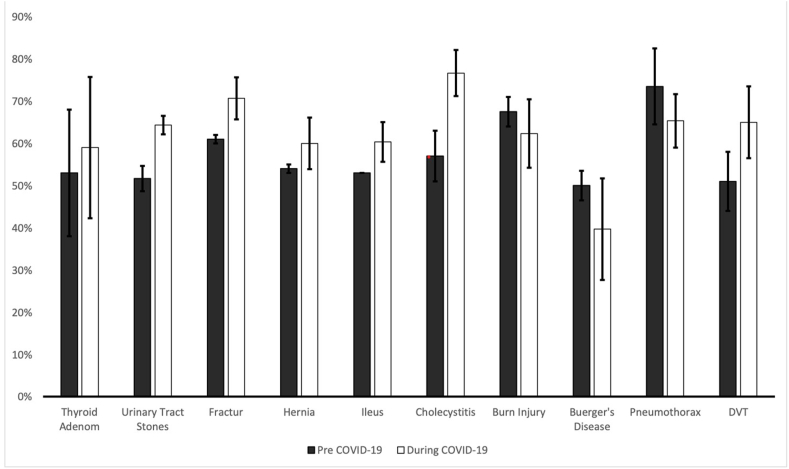
The percentage of correct MCQs for each competency before and during covid 19 showed no significant difference.

The percentage of correct answers of every period was calculated in means and SD value and compared (Fig. 2). There are no significant differences between all competencies selected.

## 4 Discussion

COVID-19 pandemic has disrupted the medical education curriculum system in particular clinical learning aspect [5]. The academic health institution has a specific scheme to solve the clerkship problem by postponing and transforming online modules and tutorials [6,7]. In our institution, surgical clerkship was combined between virtual tutorial and clinical rotation. The virtual method is used for journal reading, case-based discussion, and routine morning reports. Cycle in the outpatient clinic and ward was performed with strict protocol and personal protective equipment. The student was prohibited from getting in the emergency room, intensive care unit, covid-19 ward, and all of the red zone in the hospital. Duty hour is limited to 6 h/day.

The pandemic pushes the medical education unit to innovate the assessment method while it is an essential component of the curriculum [8]. When used properly, it can help accomplish certain curricular goals [9]. OSCE was chosen to evaluate at the end of the rotation because it is a type of assessment that has a balanced validity and reliability [10]. Online modified OSCE is a way to reduce curriculum disruption [11]. During the COVID-19 pandemic, OSCE was carried out online via the ZOOM platform. It was selected due to examiner and student familiarity and the "breakout room" feature, allowing private mini sessions (one-on-one) between host-selected participants.

Data from MCQ Score revealed no difference before and during the COVID-19 period, while OSCE scores during COVID-19 showed significantly higher than before COVID-19. A similar study with full remote learning clerkship showed higher clinical skills in COVID-19 group [12] and no difference in knowledge-based exam [12,13]. It suggested that variety and combination between the virtual and offline methods may satisfy knowledge and skills competencies. Even though clinical exposure was limited during COVID-19, our in-depth analysis of MCQ competencies found no difference. We suspect this is due to teachers dedicating more flexible time to teaching and student feedback. While OSCE in-depth analysis shown one station has a significant result than before the COVID-19 period. We believed this caused by a lower stress level student has than in-person OSCE.

## 5 Conclusion

Combining virtual platform and in-person clinical rotation is an effective surgery clerkship curriculum, particularly in pandemic covid 19. There are no different skill and knowledge results before and during the pandemic analyzed from MCQs and OSCE exam.

## Annals of medicine and surgery

The following information is required for submission. Please note that failure to respond to these questions/statements will mean your submission will be returned. If you have nothing to declare in any of these categories then this should be stated.

## Please state any sources of funding for your research

We report no involvement of any sponsor or funding body for this study.

## Ethical approval

Ethical approval has been given from the Commission of Medical Research Bioethics, Faculty of Medicine, Sultan Agung Islamic University with reference number: No.44/II/2021/Komisi Bioetik

## Consent

Written informed consent are not required for this study. Data of MCQ and OSCE scores were obtained from Evaluation Committee of Surgery Department, Sultan Agung Islamic University by sending a written permission.

## Author contribution

ES conceptualized the first draft and finalized the manuscript. FRM, AL, and ST wrote the manuscript. FRM analyzed the data. BS, AL, and ST conceived the theoritical framework. All authors read and approved the final manuscript.

## Registration of research studies

Not applicable for observational studies involving exam scores.

## Guarantor

Eko Setiawan.

## Declaration of competing interest

All authors have declared that they have no potential competing interests.
